# Is *Leadership* a Reliable Concept in Animals? An Empirical Study in the Horse

**DOI:** 10.1371/journal.pone.0126344

**Published:** 2015-05-26

**Authors:** Marie Bourjade, Bernard Thierry, Martine Hausberger, Odile Petit

**Affiliations:** 1 Unité de Recherche Interdisciplinaire Octogone, Laboratoire Cognition Communication Développement, Université Toulouse Jean Jaurès, Toulouse, France; 2 Association Takh pour le cheval de Przewalski, Station Biologique de la Tour du Valat, Arles, France; 3 Centre National de la Recherche Scientifique, Département Ecologie, Physiologie et Ethologie, Strasbourg, France; 4 Université de Strasbourg, Institut Pluridisciplinaire Hubert Curien, Strasbourg, France; 5 Laboratoire d’Ethologie Animale et Humaine, Université de Rennes 1, Centre National de la Recherche Scientifique, Rennes, France; Peking University, CHINA

## Abstract

Leadership is commonly invoked when accounting for the coordination of group movements in animals, yet it remains loosely defined. In parallel, there is increased evidence of the sharing of group decisions by animals on the move. How leadership integrates within this recent framework on collective decision-making is unclear. Here, we question the occurrence of leadership in horses, a species in which this concept is of prevalent use. The relevance of the three main definitions of leadership – departing first, walking in front travel position, and eliciting the joining of mates – was tested on the collective movements of two semi-free ranging groups of Przewalski horses (*Equus ferus przewalskii*). We did not find any leader capable of driving most group movements or recruiting mates more quickly than others. Several group members often displayed pre-departure behaviours at the same time, and the simultaneous departure of several individuals was common. We conclude that the decision-making process was shared by several group members a group movement (i.e., partially shared consensus) and that the leadership concept did not help to depict individual departure and leading behaviour across movements in both study groups. Rather, the different proxies of leadership produced conflicting information about individual contributions to group coordination. This study discusses the implications of these findings for the field of coordination and decision-making research.

## Introduction

The concept of leadership is frequently used to account for the coordination of group movements by single individuals. In gorillas (*Gorilla g*. *beringei*) and mountain baboons (*Papio h*. *ursinus*), the top-ranking male is termed as leader as its decisions appear to regulate group movements [1,2]. Packs of carnivores such as of dwarf mongooses (*Helogale parvula*), feral dogs (*Canis lupus familiaris*), and wolves (*Canis lupus*) are described to be led by top-ranking individuals [3–5]. In ungulates, leadership is commonly attributed to a single individual, usually an old female, although leaders can change according to the type of movement [6–8]. The occurrence of leaders in mammals is reported in a wide range of species, with leadership being the role of a particular category of animals [9].

Different definitions of leadership are provided in the literature, leading to different meanings and measurements. The leader is traditionally described as the animal moving in front position during travel [6,10,11], the first departing individual [4,9,12], or any animal managing to recruit partners [1,11]. All these definitions in mammals implicate that one individual is consistently recognized as the group leader over time, i.e. across multiple moving episodes. It is worth mentioning, however, that leaders are sometimes recognized by their leading behaviour at the scale of a single movement [13,14], or within pairs of individuals in experimental contexts [15–17] and in mathematical models [18,19]. The use of on-board devices for gathering animal trajectory data has also led to the measuring of leadership in terms of initiations of new trajectories by one individual, which are then followed by other group members [20–22]. All these various approaches often come up with the identification of an individual that qualifies as a leader, which can then be characterized with behavioural or personality traits [17,21,23–25], physiological needs [10,18,26,27] or individual knowledge [28,29].

To date, the different measurements of leadership have not been empirically considered within a unified study. Whether a single individual can be recognized as a leader both for its repeated movement initiations and because it steers the trajectories of group-mates is, for example, unknown. Also, theoretical research often refers to *leadership* without stating one of the above definitions [18,30,31]. This impairs efforts to evaluate not only the mechanisms but also the evolution of the means by which animals lead each other [32,33]. Similar concerns have been raised in human sciences; the leadership is defined in different and ill-connected perspectives and power and leadership are often combined under the same heading [34].

From a functional perspective, it is worth noting that the accuracy of choices made by a single individual could be lower than that resulting from decisions shared by several group members, which would incur consequences for individual fitness [35,36]. It appears that group departures are frequently preceded by pre-departure behaviours, and that collective decision-making processes still occur during movement [33,37–41]. Yet leadership has long been studied without considering the mechanisms of decision-making, and even without collecting data about departures and directional changes [6,10,11]. According to Pyritz and colleagues [42], leadership and decision-making processes must be investigated separately; leadership refers to the individuals that lead the group more often than others on the long term, whilst the decision-making process accounts for mechanisms and the participation of different group members in the coordination effort for single group movements. How leadership integrates within this framework of the decision-making process has not yet been established empirically—but see King and Sueur [43] and Sueur and Deneubourg [44] for an integrative account with mathematical modelling.

To appreciate the relevance of the leadership concept and its suitability with regard to decision-making processes, we need information about the ability of animals to recruit and coordinate their conspecifics across movements and contexts. We aimed to examine leadership in the horse since the leadership concept is particularly predominant in this species. The leader role has long been ascribed to a single individual, namely the stallion [45,46] or an old female [47,48], although some studies have come to different conclusions [25,37,49]. We studied the collective movements of two free-ranging families of Przewalski horses (*Equus ferus przewalskii*) to address Pyritz et al. [42]'s two levels of investigation, namely different measurements of leadership, and multiple-step decision-making processes within a single study design. We tested over several group movements whether specific individuals (1) consistently departed first, (2) consistently travelled in front position, or (3) were particularly efficient in eliciting joining by other group members. We then examined whether specific individuals displayed pre-departure behaviour and whether it may allow predicting leadership in the subsequent movement.

## Methods

### Study population

In March and April 2004, we followed a family of 12 Przewalski horses (referred to hereafter as the “BO group”) composed of one adult stallion (8 yrs old), five adult mares (4, 4, 5, 15 & 16 yrs old), two sub-adult females (2 yrs old) and four foals (1 yr old). The study was replicated two years later, in March and April 2006, on a family of 6 horses (referred to hereafter as the “AD group”) composed of one adult stallion (12 yrs old), three adult mares (8, 9 & 9 yrs old) and two foals (1 yr old). Both families belonged to a semi-free ranging population located in a 380-ha enclosure at *Le Villaret* (France, base camp office: 44°15’9”N, 3°26’29”E; elevation ranged from 900 to 1250 m), owned and managed by the *Takh Association*. During both study periods, the population was composed of five independent families and two all-male groups. All groups formed naturally and moved freely for vital resources without supplementary feeding or human intervention. All mares were either lactating or pregnant and lactating at the time of the study except one in the AD group. The population and study site are described in detail in Bourjade et al. [37].

### Ethics Statement

The Przewalski horse is a protected species labelled as “Endangered” on the IUCN Red list of Threatened Species (see http://www.iucnredlist.org/details/full/41763/0 for details). Our field procedure complied with current French laws and the current European directive (reference 86/609/CEE) relative to the protection of animals used for scientific purposes. According to Article 3 (definitions), this study does not qualify as an experimental procedure and therefore does not require institutional ethics approval.

### Observation procedure

To render data comparable with existing literature on group coordination, we sampled collective movements in only one functional context: that of maintenance behaviour. All movements sampled were from one foraging site to another, or to natural shelters (see [37] for details about ecological contexts). In consequence, if several individuals were found to contribute to group coordination, it will not be underpinned by mixed contexts. Group movements in response to external cues such as predator shapes (foxes, dogs…) or fleeing responses (for instance from motorbikes on a few occasions) were not considered for data collection. In the same vein, stallions frequently use “herding behaviour” during the breeding season, by which they approach group members from the back and push them away from a rival in the context of stallion rivalry [45]. This often results in group movements that do obviously not serve the aim to move from point A to point B, but rather to move away from point A. Thus, collective movements were observed outside the breeding season to limit the effect of stallion competition on family movements (88 hrs in 2004 and 120 in 2006).

Two observers approached horses on foot at a distance of about 20 m and data were collected over four consecutive hours using tape recorders. The first observer continuously recorded the occurrence of agonistic interactions and the walking order of horses when travelling. The second monitored any moving horse and recorded all the following behaviours and their time of occurrence: *pre-departure behaviour* (moving away, staying peripheral (i.e. without any neighbour within a distance of at least three horse body-lengths), following an individual that is moving away, joining a peripheral individual or pausing; see Bourjade et al. [37] for detailed definitions), *departure* (walking with the neck in a horizontal position and without stopping over a distance longer than the diameter of the group, measured in horse body-lengths), or *joining* (walking with the neck in a horizontal position and without stopping in the direction of the first moving horse over the same distance as those recorded for the departure). A collective movement began with the departure of the first moving individual and ended when the last individual arrived. It was characterized by the active walking of all individuals from the departure to the arrival sites. Collective movements were scored *a posteriori* using tape recordings of movements where at least 50% of group members simultaneously moved in the same direction.

### Analyses

We processed group movements composed of a single moving period (*single-bout movements*, see [37] for details). Specific features of the movements sampled are summarized in Table 1. We counted how many times each horse (1) took part in the pre-departure period, (2) was the first to move, or (3) was walking in front position at mid-travel time. We used the Log likelihood ratio statistic (G) with 2000 Monte-Carlo simulations and William’s correction for small samples to assess whether observed distributions arose from discrete uniform distributions [50]. We then ran pairwise comparison tests with Holm-Bonferroni corrections to test whether any individual differed from the chance value. The comparison of joining process durations—i.e. the time elapsed between departures of the first mover and those of the last mover—was used to measure the efficiency of first movers to elicit joining by the group following their respective departures.

**Table 1:** Number of collective movements recorded in the two study groups.

Types of movement	Number of records	
	BO group	AD group
Movements following the departure of a single first mover^(1)^	28	35
Movements following the departure of simultaneous first movers	14	8
Movements following a collective display of pre-departure behaviour	33	32
Movements in which a single front individual was scored at mid-travel time^(2)^	11	21
Total number of movements	42	43

^(1)^ Samples used for the analysis of departures

^(2)^ Samples used for the analysis of travels in front position.

We tested for potential relationships between the dominance rank of individuals and their tendency to lead within each group. Social hierarchies were assessed by the occurrence of unidirectional agonistic interactions. We verified their linearity through a rank order analysis carried out with Matman (Noldus): BO group, h’ = 0.60, p = 0.002; AD group, h’ = 1, p = 0.023. Dominance ranks were strongly related to age in both groups (Spearman rank correlation coefficient test: BO group, r_s_ = -0.96, p < 0.001; AD group, r_s_ = -0.97, p = 0.001), with older individuals being higher-ranked than others. Age was retained in analyses as an intrinsic feature of individuals. Non-parametric tests were used to analyse the data on leadership according to the age of individuals. All tests were two-tailed and performed with R 3.1.0 software (http://cran.r-project.org) with a level of significance setting at 0.05.

## Results

### Were consistent first movers recognizable at group departure?

**Fig 1:**
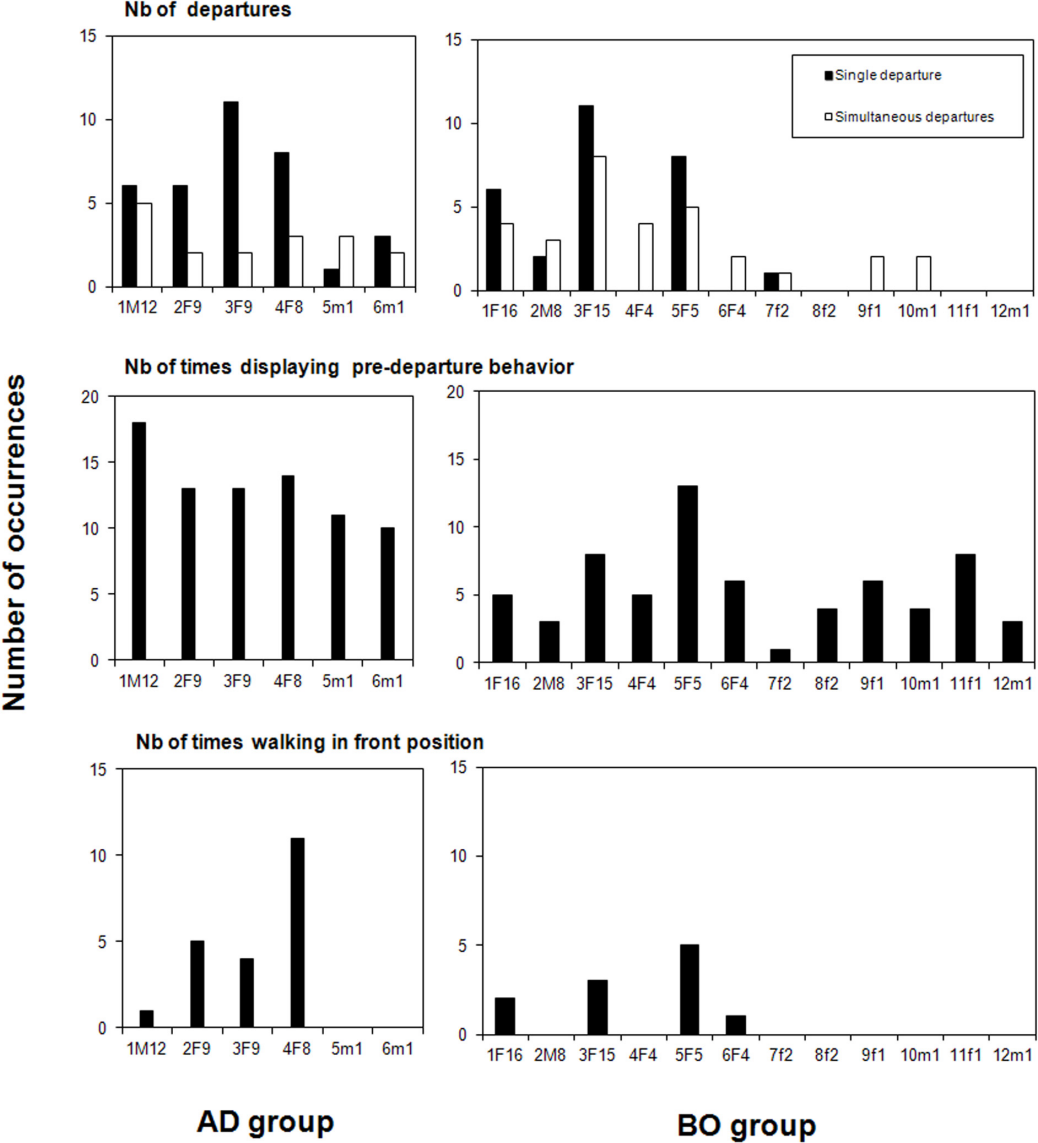
Occurrences of leadership in horse families. Leadership was defined by (a) the number of times each individual departed first; (b) the number of times each individual displayed a pre-departure behaviour (this behaviour was often performed collectively, so the number of occurrences did not match the total number of departures); (c) the number of times each individual travelled in front position. Individual codes: F: adult female, M: adult stallion, f: immature female, m: immature male; the first figure indicates the dominance rank and the second the age in years (e.g., 7f2 is a 2-yr-old female of rank 7).

Five individuals of the BO group and all individuals of the AD group were observed to depart first at least once (Fig 1). Departing first was not random in both groups (Log likelihood ratio statistic (G) with Williams’ correction: BO group, G = 58.3, df = 11, p < 0.001; AD group, G = 11.8, df = 5, p = 0.038) and was correlated with age in the BO group (Spearman test: r_s_ = 0.80, p = 0.002) but not in the AD group (Spearman test: r_s_ = 0.58, p = 0.225). Two adult mares (3F15, 5F5; Table 2) departed first above chance level in the BO group (Post-hoc tests: 3F15, p < 0.001; 5F5, p = 0.022), while no individual was found to depart first more often than expected by chance in the AD group (Post-hoc tests: p > 0.05 in all cases). When two or three horses departed at the same time, simultaneous departures were recorded. It represented 33% and 19% of the totality of movements for the BO and AD groups, respectively (Fig 1).

**Table 2:** Individual participation in the multiple-step coordination process of group movements.

		Percentages of movements for which each horse:							
Group	Individual^(2)^	Displayed pre-departure behaviour	Departed first	Walked in front position	Departed first after pre-departure behaviour	Walked in the front after pre-departure behaviour	Walked in the front after departing first and pre-departure behaviour	Displayed pre-departure behaviour before departing first	Displayed pre-departure behaviour before walking in the front
BO group									
	1F16	17.86	21.43	18.18	80.00	20.00	20.00	66.67	16.67
	2M8	10.71	7.14	0.00	66.67	0.00	0.00	100.00	0.00
	**3F15** ^**(1)**^	28.57	**39.29**	27.27	50.00	0.00	0.00	36.36	0.00
	4F4	17.86	0.00	0.00	0.00	0.00	0.00		
	**5F5** ^**(1)**^	46.43	**28.57**	**45.45**	53.85	23.08	15.38	87.50	37.50
	6F4	21.43	0.00	9.09	0.00	0.00	0.00		
	7f2	3.57	3.57	0.00	0.00	0.00	0.00	0.00	0.00
	8f2	14.29	0.00	0.00	0.00	0.00	0.00		
	9f1	21.43	0.00	0.00	0.00	0.00	0.00		
	10m1	14.29	0.00	0.00	0.00	0.00	0.00		
	11f1	28.57	0.00	0.00	0.00	0.00	0.00		
	12m1	10.71	0.00	0.00	0.00	0.00	0.00		
	*Mean*	*19*.*64*	*8*.*33*	*8*.*33*	*20*.*88*	*3*.*59*	*2*.*95*	*58*.*11*	*10*.*83*
	*SEM*	*3*.*22*	*3*.*94*	*4*.*25*	*9*.*13*	*2*.*43*	*2*.*01*	*18*.*09*	*7*.*41*
AD group									
	1M12	51.43	17.14	2.86	22.22	5.56	5.56	66.67	16.67
	2F9	37.14	17.14	14.29	15.38	15.38	7.69	33.33	33.33
	3F9	37.14	31.43	11.43	30.77	15.38	0.00	36.36	18.18
	**4F8** ^**(1)**^	40.00	22.86	**31.43**	28.57	35.71	7.14	50.00	62.50
	5m1	31.43	2.86	0.00	9.09	0.00	0.00	100.00	0.00
	6m1	28.57	8.57	0.00	20.00	0.00	0.00	66.67	0.00
	*Mean*	*37*.*62*	*16*.*67*	*10*.*00*	*21*.*01*	*12*.*01*	*3*.*40*	*58*.*84*	*21*.*78*
	*SEM*	*3*.*25*	*4*.*13*	*4*.*93*	*3*.*31*	*5*.*52*	*1*.*55*	*10*.*08*	*9*.*63*

^(1)^ Individuals who contributed significantly above chance level.

^(2)^ Individual codes: F: adult female, M: adult stallion, f: immature female, m: immature male; the first figure indicates the dominance rank and the second the age in years (e.g., 7f2 is a 2-yr-old female of rank 7)

### Did specific individuals consistently travel in front position?

Four individuals in each group were observed to travel in front position at least once (Fig 1). Travelling in front position was not random (BO group, G = 22.9, df = 11, p = 0.018; AD group, G = 25.9, df = 5, p < 0.001), and it correlated significantly with age in the BO group, but not in the AD group (Spearman test: BO group, r_s_ = 0.70, p = 0.011; AD group, r_s_ = 0.58, p = 0.225). One adult mare in each group travelled in front position significantly above chance level (Post-hoc tests: 5F5 (BO group), p = 0.016; 4F8 (AD group), p = 0.001).

### Did specific individuals elicit joining more efficiently than others?

We used the duration of the joining processes following the departure of the first movers—those departing first at least three times—to check for differences in efficiency. The mean duration of the process ± SEM was 471 ± 101 sec in the BO group, and 249 ± 82 sec in the AD group. No significant differences were found between the first movers in either group (Kruskal-Wallis test: BO group, H_2,25_ = 1.27, p = 0.531; AD group, H_4,34_ = 0.20, p = 0.995). No significant correlation was found between the age of the first mover and the duration of the subsequent joining process in both groups (Spearman test: BO group, r_s_ = 0.30, p = 0.624; AD group, r_s_ = 0.53, p = 0.280).

### Were specific individuals more involved in the pre-departure period than others?

During the 20 min time-window preceding departure, three individuals on average collectively displayed pre-departure behaviour (mean number of horses displaying pre-departure behaviour ± SEM: BO group, 3.1 ± 0.5; AD group, 3.5 ± 0.4). Their involvement in these displays (Fig 1) did not differ from a uniform distribution in the BO group (G = 18.2, df = 11, p = 0.078), nor was it significantly correlated with age (Spearman test: r_s_ = 0.38, p = 0.217). In the AD group however, adults tended to display pre-departure behaviours more often (AD group, G = 11.8, df = 5, p = 0.038), but no particular individual was found to participate in the pre-departure period more often than expected by chance (Post-hoc tests: p > 0.05 in all cases), and the correlation with age did not reach significance (Spearman test: AD group, r_s_ = 0.81, p = 0.053).

### Did pre-departure behaviour predict subsequent leadership?

Table 2 presents the percentages of movements for which each horse that displayed pre-departure behaviour departed first, walked in the front position, and/or showed both behaviours at the level of a single movement. Except for a small number of individuals, displaying pre-departure behaviour was not a reliable predictor of subsequent leading. Any individual taking part in the pre-departure period had on average 21% chance of departing first in each group, a 4% chance of subsequently walking in the front position in the BO group and 12% in the AD group, and a 3% chance of both departing first and of subsequently walking in the front position in each group. Individual differences occurred and were higher in the BO group than the AD group, as evidenced by standard errors.

Retrospectively, any first mover had displayed pre-departure behaviour prior to 58% of its departures in the BO group and 59% of its departures in the AD group. Horses walking in the front position had displayed pre-departure behaviour prior to 11% of the movements they were leading at mid-travel in the BO group, and 22% in the AD group.

## Discussion

We found no horse which could be qualified as the leader in the two groups of Przewalski horses, whatever the definition used to recognize leadership. No individual consistently moved first, elicited faster joining by group members than other first movers, or consistently travelled in front position. Most frequent first movers were responsible for 39% and 31% of departures in the BO and AD groups, respectively, and were not the ones that headed travels above chance level. Although one mare both departed first and headed travels above chance in the BO group, it was not the one that scored most departures. No horse moving first was significantly above chance level in the AD group. Finally, the display of pre-departure behaviour did not make it possible to predict subsequent leadership, as on average three individuals took part in this display, even though at least two of them-if not all three—did not depart first or head travels.

We are aware that these results suffer from some limitations due to the number of groups observed. For instance, it appears that departing first and being in a front position during movement was more frequent with increasing age in the BO group but not in the AD group. It is however important to note that age and dominance were statistically indistinguishable, as age is the main determinant of dominance rank in the horse [51]. In the same vein, we found preferential first movers in the BO group, but not in the AD group, and fewer individuals were likely to depart first or to head travels in the BO group than in the AD group. Note that the adult/young ratio of the two groups, which is known to affect spatial cohesion of young Przewalski horses [37], differed from one half of immature horses in the BO group, to one third in the AD group. It might therefore be possible that demographic structure underpinned some of the differences observed here. In this regard, the study of other groups and populations will be necessary to further document such issues. It is worth mentioning that if group size is highly variable in wild horses, the social structure itself is quite always the same [52]. Family groups observed in this study are thus representative of the typical social unit of horses living in natural conditions, and compare to other groups of Przewalksi horses in terms of ecology and social organization [53–55]. It is therefore significant that neither the oldest mares, nor the stallions contributed more to the coordination of movements than any other horses did. Thus, contrary to a very popular idea [25,45–48], age, sex and dominance seem to have very limited links with the coordination of group movements in horses (see also [56]). This finding adds critical contribution to the field given the contradictory results reported in horses so far.

One of our aims was to compare *leadership* with multiple step decision-making processes within a single study design. Crucially, first movers did not display specific signals at departure and they did not attempt to influence their conspecifics in a coercive way. This absence of motivational conflicts at the time of departure was confirmed by the fact that several individuals departed simultaneously on several occasions. It may indicate that decision-making had already occurred during the pre-departure period. Indeed, several individuals were mostly involved in the display of pre-departure behaviours. At the level of a single movement, displaying pre-departure behaviour does not predict subsequent departure as a first mover, nor subsequent travelling in front position, suggesting that the decision-making process is shared by several group members (i.e., the *Individual Axis* in Bourjade & Sueur [57]; the *Individual-Level Process* in Pyritz et al. [42]). Such an evidence of shared decision emphasizes that traditional accounts of leadership (i.e., focused on a single main individual) might not be the best way to describe group coordination or individual contributions to movement decisions, either. The display of pre-departure behaviours may facilitate overall group coordination, affect movement dynamic and decision accuracy (see [37] in this species). From an evolutionary perspective, shared decisions are relevant since they provide more accurate decision outcomes than single decisions do [35,58,59].

Evolutionary psychologists have also proposed that leadership functions might be situational in the sense that different individuals would take on the lead in certain contexts only, hence resulting in shared decisions over time through distributed leadership functions [34]. Different social niches may have favoured leadership in the course of evolution such as movement coordination, but also the regulation of internal conflicts within social groups and the management of intergroup competition. Although this study was not designed to compare leadership between functional contexts, it is likely that stallions may have fulfilled notable leadership functions in the context of intergroup encounters or predator defence [49,56], and elder individuals may possibly play a peacekeeping role within groups [51,60]. Interestingly, distributed leadership observed here does not result from various social niches in which different individuals could have emerged as leaders for specific purposes. Any individual could contribute to the pre-departure period, be the first to move, or walk in front position at mid-travel indicating that decision-making is distributed across group movements (i.e., the *Temporal Axis* in Bourjade & Sueur [57]; the *Group-Level Outcome* in Pyritz et al. [42]). Crucially, distributed leadership here is not situational; several horses contribute to group coordination within the same homogeneous context of maintenance activities over the long term.

These findings are consistent with those of Berger [49] who reports that several individuals can initiate group departures in a population of feral horses, and that changes in rank positions occur from one travel to another. A recent investigation in the domestic horse has led to this conclusion too [25]. At first sight this seems at odds with previous accounts on the role of leadership in horses [45,47,48]. However, a closer examination of these reports shows that the data supporting them did not differ that much from our own; it appears that group-members other than top-ranking/oldest ones can elicit movements or be in front position during travel in a fair proportion of the moves [45,47]. This suggests that the main discrepancy between the present findings and formerly published results in horses might reside in how the data is interpreted. Again, it raises new questions on how to define leadership. Shall an individual belonging to a group of twelve animals be called a leader if it heads a third of travels? What type of leader is an individual that often departs first but rarely heads travel? It is undeniable that some individuals contribute unequally to movement decisions. If leadership is defined in terms of departure initiation, recruitment efficiency or front position during travel, it would be still necessary to specify what proportion of group movements an individual should drive to be called a leader.

This is precisely why we wanted to confront different measurements of leadership within a unified study. There are important results derived from running these measurements on the same individuals. First, the use of one or another definition of leadership yields different leaders in each group. In the AD group, no preferential first movers were statistically distinguishable and the most frequent one was not the one that headed most travels. In the BO group, although one mare departed first significantly above chance and headed most of travels, another mare departed first more often than this individual—and was therefore the most preferential first mover. In sum, the use of variable definitions of leadership in this population led to conflicting results. This questions the reliability of these different proxies, i.e., which one is the most accurate, and even the type of information they capture, i.e., if any of them genuinely address group coordination.

In this respect, a word must be said about the meaning of leadership as behaviour or a social role. The *lean* interpretation of leadership might consider *leading* as a behaviour, and we can demonstrate that an individual leads a group at a given time and location, even fortuitously [21,61]. But in applying the term *leader* to an individual, we inadvertently jump from an objective behaviour to the concept of social role. It is obvious in many scientific reports in which researchers use the term *leaders* for individuals that successfully manage to recruit conspecifics, while they refer to those who do not as *failed initiators* [32,33]. The caveat is that a role refers to a social norm, meaning that it exists in relation to shared expectations by other group members about the consequences of the leading individual’s behaviour [62]. Notwithstanding, this *rich* interpretation of the leader role might not be within the reach of most animals [63,64]. To date, empirical studies rather suggest that first movers might have *incidental* leading effect on their conspecifics, as neither signalling nor coercive behaviour from the first movers appears to be the norm in non-primate species, e.g., in dogs [5,21]; in horses [37, 49]; in sheep [61]. However, there might have some cases of *intentional* leadership in certain animal species where one individual intends to lead and recruit group members. The minimum requirement for such an individual to qualify as an intentional leader would require it to express moving intent (e.g., pre-departure behaviour, coercive behaviour, intentional recruitment), and then depart first and head travels [57]. And it should also maintain group cohesion by returning in its group when it is not followed by others [33]. Even though returning to the group may simply be due to an individual’s assessment of the balance of its social and non-social needs, it could also be viewed as a sign of genuine intention to recruit others when it tallies with the above evidence of intentional leadership [65–67].

It then appears that our understanding of group coordination resides in the behaviour of followers [32,33,57]. Recent research on followership has shown that groups could solve coordination problems through quorum responses by which the majority of group members follow a decision after a threshold of followers has been reached [58,68]. Notably, through quorum responses, group members can increase the best outcomes (e.g., leaving when there is a predator) whilst decreasing the less adaptive ones (e.g., leaving when there is no predator), improving overall decision accuracy. More generally, when mimetic processes are at stake, for instance, the probability of each group member moving does not strictly depend on the departure of the first mover, but rather on the number of individuals already moving [57,66]. Also, followers can decide upon when to follow and positively affect decision speed, as for Pzrewalski horses that have been shown to slow down the joining process prior to ecologically demanding movements [37].

In conclusion, recent research emphasizes that the occurrence of distributed processes is likely to be the norm in animal group movements and that decision-making does occur before and after departure [12,37,39–41,69]. In such a view, *leadership* would just represent an extreme case over a continuum of decision-making processes ranging from control by a single individual to equal sharing of decisions among group members [36]. Alternatively, *Leadership* and decision-making have been thought to be two distinct levels of group coordination that are not mutually exclusive [42]. In this respect, the present results enable us to express the need for caution when using *leadership* as a way of studying animal group movements. In its current state, the concept of leadership proved unreliable in the horse, a species where it has been used for decades without addressing the nature of the decision-making process. This study should allow future research in the field of group coordination to avoid this pitfall.

Finding that the concept of leadership does not satisfactorily account for the behaviour of two groups of horses does not disqualify this concept in all mammals. Whether it should be abandoned or made operational is still under debate [32,33,42]. Despite the widespread use of the leadership concept in the literature, it should be stressed that no study has so far quantitatively demonstrated that certain individuals consistently play the leader role in the group movements of animals. At present, studying the recruitment processes through which individuals come to follow others appears as a more promising approach than focusing on single individuals to uncover the mechanisms through which mammals reach collective decisions.

## References

[1] Byrne RW, Whiten A, Henzi SP (1990) Social relationships of mountain baboons: Leadership and affiliation in a non-female-bonded monkey. Am J Primatol. 20: 313–329. 10.1002/ajp.1350200409 32075347

[2] Schaller GE. The mountain gorilla: Ecology and behavior Oxford: The University of Chicago Press; 1963.

[3] Holekamp KE, Boydston EE, Smale L. Group travel in social carnivores. In: Boinski S, Garber PA, editors. On the move: How and why animals travel in groups Chicago, US: University of Chicago Press; 2000 p. 587–627.

[4] Peterson RO, Jacobs AK, Drummer TD, Mech LD, Smith DW (2002) Leadership behavior in relation to dominance and reproductive status in gray wolves, *Canis lupus* . Can J Zool. 80: 1405–1412.

[5] Bonanni R, Cafazzo S, Valsecchi P, Natoli E (2010) Effect of affiliative and agonistic relationships on leadership behaviour in free-ranging dogs. Anim Behav 79: 981–991. 10.1016/j.anbehav.2010.02.021

[6] Dumont B, Boissy A, Achard C, Sibbald AM, Erhard HW (2005) Consistency of animal order in spontaneous group movements allows the measurement of leadership in a group of grazing heifers. Appl Anim Behav Sci 95: 55–66. 10.1016/j.applanim.2005.04.005

[7] Réale D, Festa-Bianchet M (2003) Predator-induced natural selection on temperament in bighorn ewes. Anim Behav 65: 463–470. 10.1006/anbe.2003.2100 11082229

[8] Reinhardt V (1983) Movement orders and leadership in a semi-wild cattle herd. Behaviour 83: 251–264. 10.1163/156853983X00183

[9] Boinski S, Garber PA. On the move: How and why animals travel in groups Chicago, US: University of Chicago Press; 2000.

[10] Fischhoff IR, Sundaresan SR, Cordingley J, Larkin HM, Sellier M-J, Rubenstein DI (2007) Social relationships and reproductive state influence leadership roles in movements of plains zebra, *Equus burchellii* . Anim Behav 73: 825–831. 10.1016/j.anbehav.2006.10.012

[11] King AJ, Douglas CMS, Huchard E, Isaac NJB, Cowlishaw G (2008) Dominance and affiliation mediate despotism in a social primate. Curr Biol 18: 1833–1838. 10.1016/j.cub.2008.10.048 19026539

[12] Leca J-B, Gunst N, Thierry B, Petit O (2003) Distributed leadership in semifree-ranging white-faced capuchin monkeys. Anim Behav 66: 1045–1052. 10.1006/anbe.2003.2276

[13] Krause J, Hoare D, Krause S, Hemelrijk CK, Rubenstein DI (2000) Leadership in fish shoals. Fish Fish 1: 82–89.

[14] Lamprecht J (1996) What makes an individual the leader of its group? An evolutionary concept of distance regulation and leadership. Soc Sci Inf 35: 595–617. 10.1177/053901896035004001

[15] Harcourt JL, Ang TZ, Sweetman G, Johnstone RA, Manica A (2009) Social feedback and the emergence of leaders and followers. Curr Biol 19: 248–252. 10.1016/j.cub.2008.12.051 19185497

[16] Nakayama S, Johnstone RA, Manica A (2012) Temperament and hunger interact to determine the emergence of leaders in pairs of foraging fish. PloS One 7: e43747 10.1371/journal.pone.0043747 22952753 PMC3430686

[17] Nakayama S, Harcourt JL, Johnstone RA, Manica A (2012) Initiative, personality and leadership in pairs of foraging fish. PloS One 7: e36606 10.1371/journal.pone.0036606 22567168 PMC3342251

[18] Rands SA, Cowlishaw G, Pettifor RA, Rowcliffe JM, Johnstone RA (2003) Spontaneous emergence of leaders and followers in foraging pairs. Nature 423: 432–434. 12761547 10.1038/nature01630

[19] Rands SA, Cowlishaw G, Pettifor RA, Rowcliffe JM, Johnstone RA (2008) The emergence of leaders and followers in foraging pairs when the qualities of individuals differ. BMC Evol Biol 8: 51 10.1186/1471-2148-8-51 18282297 PMC2276478

[20] Nagy M, Ákos Z, Biro D, Vicsek T (2010) Hierarchical group dynamics in pigeon flocks. Nature 464: 890–893. 10.1038/nature08891 20376149

[21] Ákos Z, Beck R, Nagy M, Vicsek T, Kubinyi E (2014) Leadership and path characteristics during walks are linked to dominance order and individual traits in dogs. PLoS Comput Biol 10: e1003446 10.1371/journal.pcbi.1003446 24465200 PMC3900374

[22] Šárová R, Špinka M, Panamá JLA, Šimeček P (2010) Graded leadership by dominant animals in a herd of female beef cattle on pasture. Anim Behav 79: 1037–1045.

[23] Kurvers RH, Eijkelenkamp B, van Oers K, van Lith B, van Wieren SE, Ydenberg RC, et al (2009) Personality differences explain leadership in barnacle geese. Anim Behav 78: 447–453. 10.1111/j.1365-2656.2008.01488.x 19302127

[24] Johnstone RA, Manica A (2011) Evolution of personality differences in leadership. Proc Natl Acad Sci 108: 8373–8378. 10.1073/pnas.1102191108 21536882 PMC3100967

[25] Krueger K, Flauger B, Farmer K, Hemelrijk C (2014) Movement initiation in groups of feral horses. Behav Proc 103: 91–101. 10.1016/j.beproc.2013.10.007 24220794

[26] Krause J, Bumann D, Todt D (1992) Relationship between the position preference and nutritional state of individuals in schools of juvenile roach (*Rutilus rutilus*). Behav Ecol Sociobiol 30: 177–180.

[27] Sueur C, Deneubourg J-L, Petit O, Couzin ID (2010) Differences in nutrient requirements imply a non-linear emergence of leaders in animal groups. PLoS Comput Biol 6: e1000917 10.1371/journal.pcbi.1000917 20824127 PMC2932680

[28] Reebs SG (2000) Can a minority of informed leaders determine the foraging movements of a fish shoal? Anim Behav 59: 403–409. 10675263 10.1006/anbe.1999.1314

[29] Faria JJ, Dyer JRG, Tosh CR, Krause J (2010) Leadership and social information use in human crowds. Anim Behav 79: 895–901. 10.1016/j.anbehav.2009.12.039

[30] Couzin ID, Krause J, Franks NR, Levin SA (2005) Effective leadership and decision-making in animal groups on the move. Nature 433: 513–516. 15690039 10.1038/nature03236

[31] King AJ, Johnson DDP, Van Vugt M (2009) The origins and evolution of leadership. Curr Biol 19: R911–R916. 10.1016/j.cub.2009.07.027 19825357

[32] King AJ (2010) Follow me! I’m a leader if you do; I’m a failed initiator if you don’t? Behav Proc 84: 671–674. 10.1016/j.beproc.2010.03.006 20350591

[33] Petit O, Bon R (2010) Decision-making processes: The case of collective movements. Behav Proc 84: 635–647. 10.1016/j.beproc.2010.04.009 20435103

[34] Van Vugt M, Hogan R, Kaiser RB (2008) Leadership, followership, and evolution: Some lessons from the past. Am Psychol 63: 182 10.1037/0003-066X.63.3.182 18377108

[35] List C (2004) Democracy in animal groups: A political science perspective. Trends Ecol Evol 19: 168–169. 16701250 10.1016/j.tree.2004.02.004

[36] Conradt L, Roper TJ (2005) Consensus decision making in animals. Trends Ecol Evol 20: 449–456. 16701416 10.1016/j.tree.2005.05.008

[37] Bourjade M, Thierry B, Maumy M, Petit O (2009) Decision-making in Przewalski horses (*Equus ferus przewalskii*) is driven by the ecological contexts of collective movements. Ethology 115: 321–330. 10.1111/j.1439-0310.2009.01614.x

[38] Kummer H. Social organization of hamadryas baboons: A field study 1st ed. Chicago, US: The University of Chicago Press; 1968.

[39] Ramseyer A, Thierry B, Boissy A, Dumont B (2009) Decision-making processes in group departures of cattle. Ethology 115: 948–957.

[40] Ramseyer A, Boissy A, Dumont B, Thierry B (2009) Decision making in group departures of sheep is a continuous process. Anim Behav 78: 71–78.

[41] Sueur C, Petit O (2008) Shared or unshared consensus decision in macaques? Behav Proc 78: 84–92. 10.1016/j.beproc.2008.01.004 18281161

[42] Pyritz LW, King AJ, Sueur C, Fichtel C (2011) Reaching a consensus: Terminology and concepts used in coordination and decision-making research. Int J Primatol 32: 1268–1278. 10.1007/s10764-011-9524-9 22207769 PMC3228941

[43] King AJ, Sueur C (2011) Where next? Group coordination and collective decision making by primates. Int J Primatol 32: 1245–1267. 10.1007/s10764-011-9524-9 PMC3228941 22207769

[44] Sueur C, Deneubourg J-L (2011) Self-organization in primates: Understanding the rules underlying collective movements. Int J Primatol 32: 1413–1432.

[45] Feist JD, McCullough DR (1976) Behavior patterns and communication in feral horses. Z Tierpsychol 41: 337–371. 983427 10.1111/j.1439-0310.1976.tb00947.x

[46] Ozogány K, Vicsek T (2014) Modeling the emergence of modular leadership hierarchy during the collective motion of herds made of harems. J Stat Phys: 1–19. 10.1007/s10955-014-1131-7

[47] Tyler SJ (1972) The behaviour and social organization of the New Forest ponies. Anim Behav Monogr 5: 87–196.

[48] Welsh DA (1975) Population, behavioural and grazing ecology of the horses of Sable Island, Nova Scotia Thesis (Ph. D.)–Dalhousie University.

[49] Berger J (1977) Organizational systems and dominance in feral horses in the Grand Canyon. Behav Ecol Sociobiol 2: 131–146.

[50] Sokal RR, Rohlf FJ. Biometry: The principles and practice of statistics in biological research San Francisco: Freeman; 1995.

[51] Fureix C, Bourjade M, Henry S, Sankey C, Hausberger M (2012) Exploring aggression regulation in managed groups of horses *Equus caballus* . Appl Anim Behav Sci 138: 216–228. 10.1016/j.applanim.2012.02.009

[52] Linklater WL (2000) Adaptive explanation in socio-ecology: lessons from the Equidae. Biol Rev 75: 1–20. 10740891 10.1017/s0006323199005411

[53] King SRB, Gurnell J (2010) Effects of fly disturbance on the behaviour of a population of reintroduced Przewalski horses (*Equus ferus przewalskii*) in Mongolia. Appl Anim Behav Sci 125: 22–29. 10.1016/j.applanim.2010.03.006

[54] Souris A-C, Kaczensky P, Julliard R, Walzer C (2007) Time budget, behavioral synchrony and body score development of a newly released Przewalski’s horse group *Equus ferus przewalskii*, in the Great Gobi B Strictly Protected Area in SW Mongolia. Appl Anim Behav Sci 107: 307–321. 10.1016/j.applanim.2006.09.023 22064904 PMC3207227

[55] Xia C, Cao J, Zhang H, Gao X, Yang W, Blank D (2014) Reintroduction of Przewalski’s horse (*Equus ferus przewalskii*) in Xinjiang, China: The status and experience. Biol Conserv 177: 142–147. 10.1016/j.biocon.2014.06.021

[56] Feh C. Relationships and communication in socially natural horse herds In: Mills DS, McDonnell SM, editors. The Domestic Horse: The origins, development and management of its behaviour Cambridge, UK: Cambridge University Press; 2005.

[57] Bourjade M, Sueur C (2010) Shared or unshared consensus for collective movement? Towards methodological concerns. Behav Proc 84: 648–652. 10.1016/j.beproc.2010.02.027 20211230

[58] Sumpter DJT, Pratt SC (2009) Quorum responses and consensus decision making. Philos Trans R Soc Lond B 364: 743–753. 10.1098/rstb.2008.0204 19073480 PMC2689713

[59] King AJ, Cowlishaw G (2007) When to use social information: The advantage of large group size in individual decision making. Biol Lett 3: 137–139. 10.1098/rsbl.2007.0017 17284400 PMC2104485

[60] Bourjade M, de Boyer des Roches A, Hausberger M (2009) Adult-young ratio, a major factor regulating social behaviour of young: A horse study. PLoS ONE 4: e4888 10.1371/journal.pone.0004888 19293930 PMC2654111

[61] Pillot MH, Gautrais J, Gouello J, Michelena P, Sibbald A, Bon R (2010) Moving together: Incidental leaders and naïve followers. Behav Proc 83: 235–241. 10.1016/j.beproc.2009.11.006 19931601

[62] Hinde RA. Biological bases of human social behaviour New-York: McGraw-Hill; 1974.

[63] Tomasello M, Carpenter M (2007) Shared intentionality. Dev Sci 10: 121–125. 17181709 10.1111/j.1467-7687.2007.00573.x

[64] Tomasello M, Carpenter M, Call J, Behne T, Moll H (2005) Understanding and sharing intentions: The origins of cultural cognition. Behav Brain Sci 28: 675–691. 10.1017/S0140525X05000129 16262930

[65] Meunier H, Deneubourg J-L, Petit O (2008) How many for dinner? Recruitment and monitoring by glances in capuchins. Primates 49: 26–31. 17646923 10.1007/s10329-007-0055-0

[66] Petit O, Gautrais J, Leca J-B, Theraulaz G, Deneubourg J-L (2009) Collective decision-making in white-faced capuchin monkeys. Proc R Soc B 276: 3495–3503. 10.1098/rspb.2009.0983 19605395 PMC2817197

[67] Sueur C, Petit O (2010) Signals use by leaders in *Macaca tonkeana* and *Macaca mulatta*: group-mate recruitment and behaviour monitoring. Anim Cogn 13: 239–248. 10.1007/s10071-009-0261-9 19597854

[68] Wolf M, Kurvers RHJM, Ward AJW, Krause S, Krause J (2013) Accurate decisions in an uncertain world: collective cognition increases true positives while decreasing false positives. Proc R Soc Lond B 280: 20122777 10.1098/rspb.2012.2777 23407830 PMC3574371

[69] Stueckle S, Zinner D (2008) To follow or not to follow: Decision making and leadership during the morning departure in chacma baboons. Anim Behav 75: 1995–2004.

